# Comparative Mid- to Long-Term Effects of Bariatric Surgery Versus Medical/Lifestyle Management in Type 2 Diabetes Mellitus: A Network Meta-Analysis of Randomized Controlled Trials

**DOI:** 10.1007/s11695-025-08451-x

**Published:** 2026-01-23

**Authors:** Aycan Celik Esmer, Serdar Sever, Burak Kundakci

**Affiliations:** 1 Faculty of Health Sciences, Internal Medicine Nursing Department, Usak University, Uşak, Turkey; 2 Faculty of Health Sciences, Surgical Nursing Department, Usak University, Uşak, Turkey; 3 Centre for Musculoskeletal Research, Faculty of Biology, Medicine and Health, University of Manchester, Manchester, UK; 4 School of Medicine and Population Health, Sheffield Centre for Health and Related Research (SCHARR), University of Sheffield, Sheffield, UK; 5 Department of Physiotherapy and Rehabilitation, Faculty of Health Sciences, Ordu University, Ordu, Turkey

**Keywords:** Type 2 diabetes, Bariatric surgery, HbA1c levels, T2DM remission, Cardiometabolic profiles, Network meta-analysis

## Abstract

A network meta-analysis of randomized controlled trials (RCTs) was conducted to establish the ranking of various bariatric surgeries in mid- and long-term follow-up durations for people with type 2 diabetes mellitus (T2DM), based on T2DM remission, HbA1c levels, and cardiometabolic outcomes. We searched PubMed, Embase, Medline, and Cochrane Central databases. A common comparator was identified between interventions to develop a network. Mean difference (MD) and 95% confidence interval (CI) were estimated between interventions. Direct and indirect evidence were pooled using the random-effect model. Eleven RCTs and four bariatric surgeries, including Roux-en-Y gastric bypass (RYGB), laparoscopic-adjustable gastric banding (LAGB), biliopancreatic diversion without duodenal switch (BPD), sleeve gastrectomy (SG), and medical/lifestyle treatment (MT) as a comparator group, were included. RYGB, LAGB, BPD, SG, as compared with MT, were all significantly associated with the remission of T2DM and HbA1c levels. While BPD is the best one for remission (MD: -5.14 [95% CI -7.33 to -2.96]), HbA1c (MD: -31.75 [95% CI -37.73 to -25.77]), the evidence is sparse. RYGB was the most effective surgery type for BMI, TG, and DBP for the mid-term follow-up period. Moderate mid- and long-term effects of T2DM remission and HbA1c levels were observed after all surgical procedures. However, BPD appears to be promising for achieving long-term diabetes remission, HbA1c levels, and cardiometabolic profiles. RYGB is the most favourable option to manage HbA1c levels and BMI. The effects of other surgical types on cardiometabolic profiles are inconclusive and require further research.

**Supplementary Information:**

The online version contains supplementary material available at 10.1007/s11695-025-08451-x.

## Key Points

RYGB, LAGB, BPD, SG were all significantly associated with the remission of T2DM and HbA1c levels as compared with MT.

In long-term follow-up, BPD appears to be the most effective surgery for achieving diabetes remission, HbA1c levels, and cardiometabolic profiles, however evidence is sparse.

Bariatric surgery has a favourable effect on diabetes related outcomes.

**Supplementary Information:**

The online version contains supplementary material available at 10.1007/s11695-025-08451-x.

## Introduction

 The prevalence of diabetes is increasing rapidly around the world. Due to this trend, the number of diabetic patients is expected to be approximately 366 million in 2030 [1]. Type 2 diabetes mellitus (T2DM) and complications are associated with increased health care expenditures and reduced quality of life, which pose a burden on society and reduce productivity [2, 3]. Patients with obesity (BMI > 30 kg/m^2^) are more likely to progress to T2DM, and around 60% of patients with T2DM are obese [4, 5].

Medical therapies and lifestyle interventions are often preferred for the management of T2DM; however, it is hard to maintain diabetes remission for the long term based on clinical trials [6]. Achievement and sustainment of clinically meaningful reductions in weight is difficult with medical therapies and lifestyle interventions in patients with obesity and T2DM since behaviour change is mostly dependent on long-term adherence [7]. In addition, some example interventions coupling the pharmacotherapy with lifestyle modifications only show modest weight loss [8]. Although around 6% weight loss was observed in a previous trial, there was no clear impact on cardiovascular disease risk reduction [9]. Higher weight loss may be necessary to facilitate improvement in the risk of cardiovascular disease [6].

Bariatric surgery, also known as metabolic surgery, regarded as the most effective intervention for obesity and primarily aims to reduce weight significantly, sustain this reduction, and thereby improve and lead to remission of obesity related conditions, specifically T2DM [8, 10, 11]. The procedures, including Roux-en-Y gastric bypass (RYGB), sleeve gastrectomy (SG), laparoscopic adjustable gastric banding (LAGB), and biliopancreatic diversion (BPD), not only facilitate substantial weight loss but also provide additional health benefits, including improvements in cardiometabolic conditions such as hypertension and hyperlipidaemia [8, 10–13]. Bariatric surgery is also recommended to obese patients (BMI > 30 kg/m^2^) with T2DM in the recent joint statement of the American Society for Metabolic and Bariatric Surgery and the International Federation for the Surgery of Obesity and Metabolic Disorders [14].

A previous meta-analysis attempted to measure the effectiveness of bariatric surgery in the short term, including mostly studies with < 2 years of follow-up [15]. Another meta-analysis investigated the long-term outcomes for non-obese patients, including observational studies with a degree of heterogeneity [16]. However, there is a scarcity of evidence measuring the effectiveness of RCTs based on different types of surgery methods to understand which one has the most favourable impact for the mid and long-term outcomes for T2DM patients. Therefore, our network meta-analysis addresses this issue by focusing only on studies with ≥ 2 years outcomes. Furthermore, we aim to compare diabetes remission and changes in cardiometabolic outcomes in patients receiving different types of bariatric surgery to provide a new understanding for healthcare professionals based on the latest evidence.

## Methods

### Literature Search

This network meta-analysis was designed according to the PRISMA Extension Statement for Reporting of Systematic Reviews Incorporating Network Meta-analyses of Health Care Interventions: Checklist and Explanations [17]. For transparency, the protocol of this review was registered at the International Prospective Register of Systematic Reviews (PROSPERO) database (Registration number: CRD42024524446).

A systematic search strategy was performed in several databases, which include the following: PubMed, EMBASE, MEDLINE, and COCHRANE Central database. Databases were searched without time limitations. The database search was conducted from the inception of each database to June 1, 2024. Only studies reported in the English and Turkish languages were included in the review because of the limitations of translation resources. The search was undertaken using a combination of Medical Subject Headings (MeSH) that included all sub-terms and sub-headings, free texts, and truncation and Boolean operators. The following research terms and operators were used: Type 2 diabet* OR noninsulin depend$ diabet$ AND bariatric surger* OR gastric surger* OR metabolic surger* OR bariatric surgical procedur*. A search strategy designed for MEDLINE is given in Supplementary File 1: Search strategy. Reference lists of the existing studies were scanned to identify any additional studies.

## Eligibility and Exclusion Criteria

Studies were eligible if they were:

(i) randomized controlled trials,
(ii) had at least 2 years of follow-up, (iii) included people with T2DM,
(iii) investigated the impact of bariatric surgery,
(iv) investigated a comparator medical/lifestyle treatment for T2DM, and
(v) reported diabetes outcomes.

Primary outcomes were T2DM remission and HbA1c levels. Secondary outcomes were cardiometabolic outcomes, including BMI, blood pressure [systolic blood pressure (SBP), diastolic blood pressure (DBP)], and lipid profiles [triglyceride (TG), total cholesterol (TCHO), and low-density lipoprotein (LDL)]. Mid-term and long-term follow-up periods were defined as follows: mid-term was defined as any follow-up between two and less than five years (using the timepoint closest to five years), and long-term was defined as five years or longer (using the longest available follow-up).

The following exclusion criteria was applied:

(i) studies that do not report any of our outcomes of interest,
(ii) all non-RCTs such as cross-sectional studies and cohort studies to increase the quality of the study,
(iii) qualitative studies that cannot provide any data for this research question which needs to be answered in a quantitative form,
(iv) studies using of duplicate data set, not having raw data available,
(v) studies including literature review, systematic review, review articles and abstracts that do not supply enough information to evaluate the study.

## Study Selection and Data Extraction

All studies identified through the search process were initially screened and reviewed by examining their titles and abstracts to identify relevant studies. This process was conducted independently by two reviewers (ACE, SS). The full texts of potentially relevant studies were then retrieved and reviewed by the same two reviewers. Any disagreements were resolved through discussion with a third reviewer (BK). For RCTs, data extraction was performed using a standardised, pre-piloted template. Data extraction involved two reviewers (ACE, SS) working independently, and any discrepancies were resolved through discussion with the third reviewer (BK).

The following characteristics were extracted: author, year, country, study design/methods, surgery, study aim, the characteristics of the study sample (e.g. sample size, age range, diabetes duration), follow-up (months), and main results.

When required data were not published, the authors were contacted for additional information. If no response was received, missing data were estimated using alternative values reported in the manuscripts (e.g. standard deviations were calculated from standard errors, 95% confidence intervals, and sample sizes, as recommended in the Cochrane Handbook.

## Risk of Bias Assessment

We used the Cochrane Collaboration’s Risk of Bias tool [18] to assess the methodological quality of the included studies. Two independent reviewers (ACE, SS) classified studies as high, low, or unclear risk of bias based on the following domains: random sequence generation, allocation concealment, blinding of participants and personnel, blinding of outcome assessment, incomplete outcome data, selective outcome reporting and other source of bias. Discrepancies were resolved first by discussion, followed by consulting with a third reviewer (BK) if needed. 

### Statistical Analysis

A network meta-analysis (NMA) was conducted for the analysis. A common comparator was identified between interventions to develop a network. For continuous data, mean difference (MD) of the change score and 95% confidence interval (CI) were estimated between interventions, while for dichotomous data, odds ratios (OR) were calculated. Units were converted as needed using an online calculator [19] to calculate the MD and ensure consistency across studies. Direct and indirect evidence were pooled using the random-effects model. The NMA was implemented in STATA using the “network” package.

We conducted analyses based on different surgical interventions. In addition, we had planned to conduct subgroup analyses based on patient characteristics such as age, BMI, and sample size, as well as sensitivity analyses based on risk of bias, imputed data, and restricting the primary outcome to studies using a uniform definition of complete remission. However, due to the limited number of included studies, these analyses could not be conducted.

Similarly, Egger’s test was planned to assess publication bias but was not performed, as fewer than 10 studies were included.

### Assessment of Transitivity and Consistency

The assumption of transitivity was assessed by examining the distribution of potential effect modifiers, including age, BMI, and diabetes duration, across all treatment comparisons. Consistency between direct and indirect evidence was evaluated using both global (design-by-treatment interaction) and local (node-splitting) approaches.

## Patient and Public Involvement

We involved two patients with a history of bariatric surgery throughout the process, from outcome prioritization to interpretation and dissemination of results.

## Results

### Study Selection

**Fig. 1:**
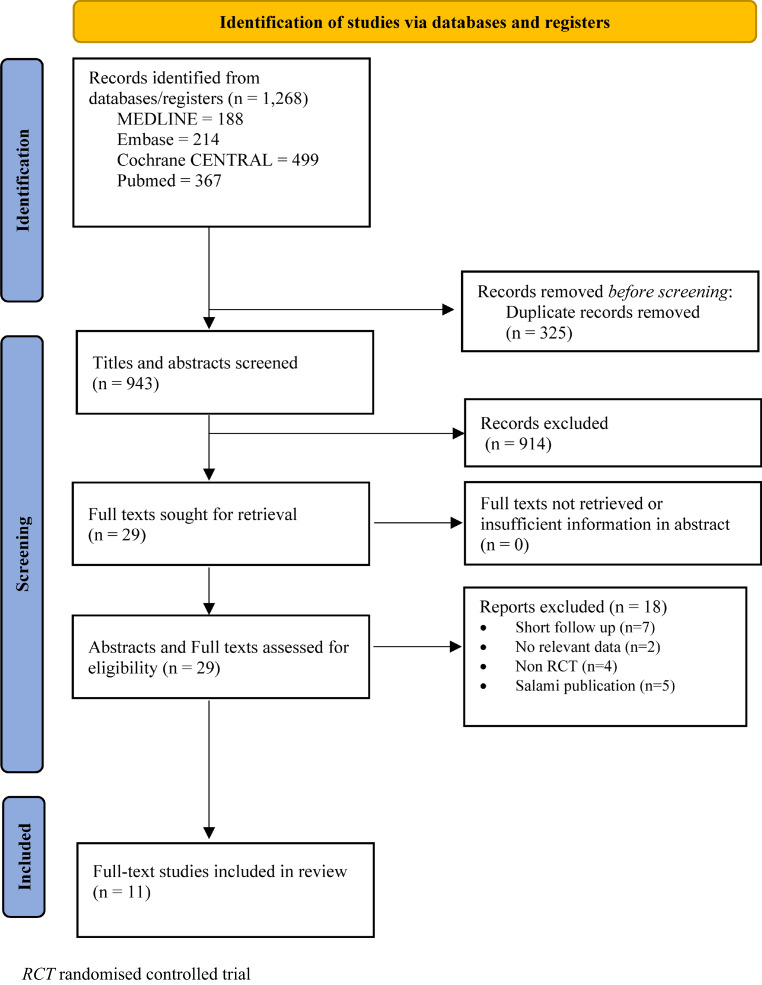
Flow diagram

A total of 1268 citations were identified from database searches. After 325 duplicates were removed, 943 titles and abstracts were screened against the inclusion and exclusion criteria, which led to the exclusion of 914 articles. The full texts of the remaining 29 papers were screened for eligibility, and 18 articles were excluded because they did not meet the inclusion criteria. After evaluating articles according to selection, 11 eligible RCTs (see Fig. 1) that met the inclusion criteria were finally included.

###  Study Characteristics 

 Eleven RCTs included a total of 740 people with T2DM. A total of 4 surgical procedures were evaluated in the primary studies, which included Roux-en-Y gastric bypass (RYGB, 8 studies) [11, 13, 20–25], laparoscopic adjustable gastric banding (LAGB, 4 studies) [11, 12, 26, 27], biliopancreatic diversion (BPD, 1 study) [23], sleeve gastrectomy (SG, 3 studies) [13, 22, 24]. The number of people with T2DM in each intervention arm was 238 in RYGB, 105 in SG, 90 in LAGB, and 20 in BPD, while the medical treatment arm included 287 people with T2DM.

 Of the 11 eligible studies identified, seven were conducted in the USA [11, 13, 21, 22, 24, 25, 27], two in Australia [12, 26], one in Singapore [20], and one in Italy [23]. The mean diabetes duration of the study population ranged from 1,5 to 10,6 years. Study follow-up ranged from 2 to 10 years. The mean BMI of the study population was 35>kg/m^2^, except for the three RCTs by Cheng et al. 2022 [20], Qi et al. 2023 [12], and Ikramuddin et al. 2015 [28] which were 35m^2^. The baseline characteristics of people with T2DM in each treatment arm are summarised in Table 1.

**Table 1:** Characteristics of included studies

Author, year	Country	Interventions vs. control	Sample size	Age mean	BMI	Outcomes	T2DM remission criteria
Aminian et al. 2021 [13]	USA	RYGB SG MT	104	48.9	36.5	HbA1c, body weight, LDL, HDL, triglycerides, SBP, DBP	NR
Cheng et al. 2022 [20]	Singapore	RYGB MT	26	44.3	29.4	T2DM remission, HbA1c, body weight, LDL, HDL, triglycerides, SBP, DBP	Complete diabetes remission defined as HbA1c ≤ 6% (≤ 42 mmol/mol) without the use of glucose-lowering medication at 12 months post-intervention and beyond.
Courcoulas et al. 2020 [11]	USA	RYGB LAGB MT	61	47.3	35.7	T2DM remission, HbA1c, body weight, LDL, HDL, triglycerides, SBP, DBP	Complete remission was defined as absence of medications with HbA1c < 5.7% and FPG ≤ 100 mg/dL
Daminian Qi et al. [12]	Australia	LAGB MT	41	54.0	29.5	T2DM remission, HbA1c, body weight, LDL, HDL, triglycerides, SBP, DBP, microvascular, macrovascular complications	Diabetes remission as HbA1c < 6.5% (48mmol/mol) without use of glucose-lowering medication for the preceding 3 months.
Dixon et al. 2008 [26]	Australia	LAGB MT	60	46.9	37.1	T2DM remission, HbA1c, body weight, LDL, HDL, triglycerides, SBP, DBP, total cholesterol	Remission of type 2 diabetes (fasting glucose level < 126 mg/dL [7.0 mmol/L] and glycated hemoglobin [HbA1c] value < 6.2% while taking no glycemic therapy
Ikramuddin et al. [21]	USA	RYGB MT	119	49.0	34.6	T2DM remission, HbA1c, body weight, LDL, HDL, triglycerides, SBP, DBP, total cholesterol	Diabetes remission is defined as an HbA1c level of less than 6.0%.
Kashyap et al. 2013 [22]	USA	RYGB SG MT	54	48.4	36.1	HbA1c, body weight, LDL, HDL, triglycerides	NR
Mingrone et al. 2012 [10]	Italy	RYGB BPD MT	60	43.4	> 35	T2DM remission, HbA1c, body weight, LDL, HDL, triglycerides, SBP, DBP, total cholesterol	HbA1C < 6.5%, FPG < 100 mg/dl, no glycemic medications.
Schauer et al. 2014 [24]	USA	RYGB SG MT	137	48.6	36	T2DM remission, HbA1c, body weight, LDL, HDL, triglycerides, SBP, DBP, total cholesterol	HbA1c < 6.0% without diabetes medications
Simonson et al. 2018 [25]	USA	RYGB MT	38	51.7	36.3	HbA1c, body weight, LDL, HDL, triglycerides, SBP, DBP, total cholesterol	NR
Simonson et al. 2019 [27]	USA	LAGB MT	40	51.3	36.5	HbA1c, body weight, LDL, HDL, triglycerides, SBP, DBP, total cholesterol	NR

###  Network Map

**Fig. 2:**
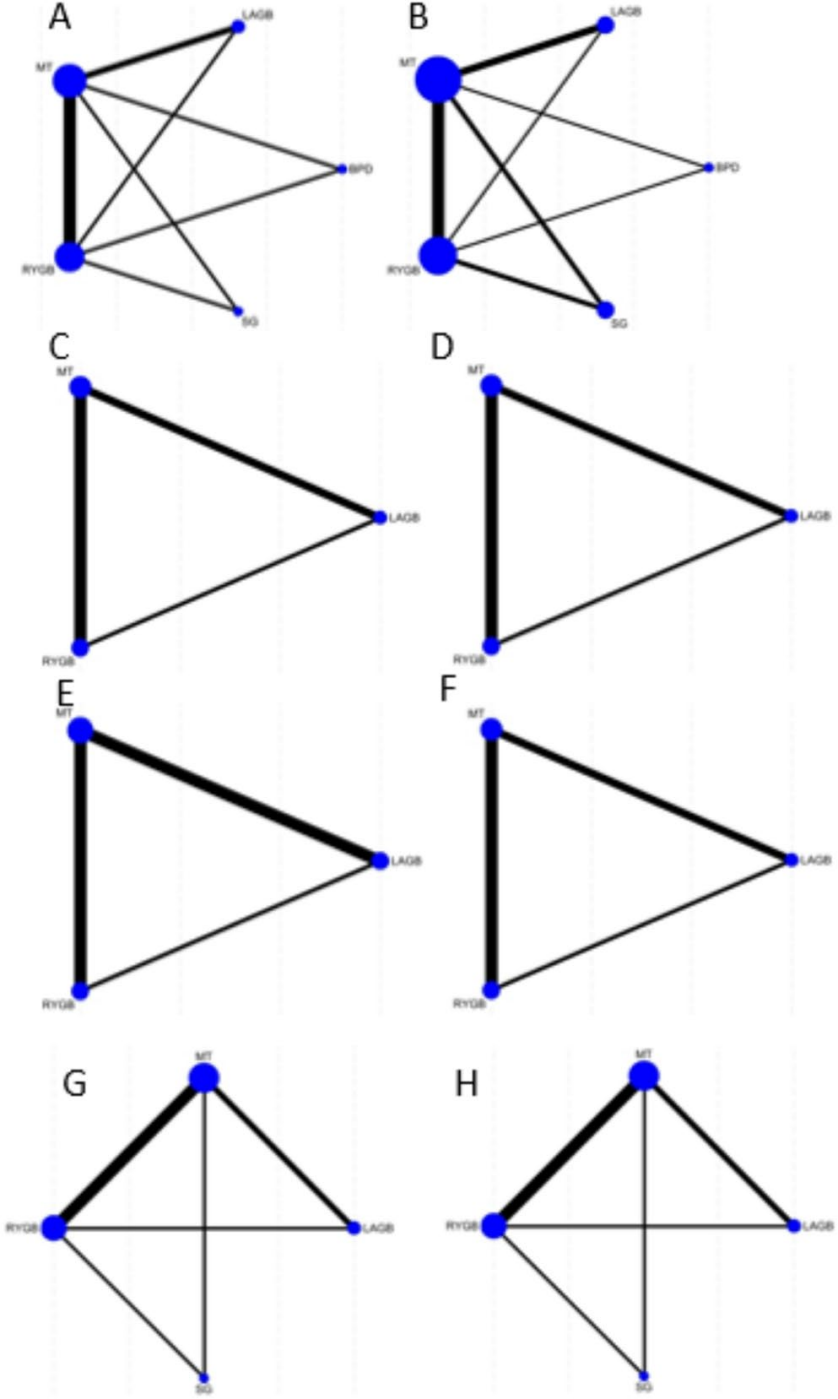
Network maps. **A**: Remission of T2DM; type 2 diabetes, **B**: HbA1c: Haemoglobin A1c, **C**: BMI; Body Mass Index, **D**: TG; Triglyceride, **E** TC; Total Cholesterol,** F**: LDL; Low-density Lipoprotein, G: SBP; Systolic Blood Pressure, H: DBP; Diastolic Blood Pressure

Figure 2 illustrates the generated network maps among bariatric surgery interventions for all outcomes.

###  Risk of Bias 

**Fig. 3:**
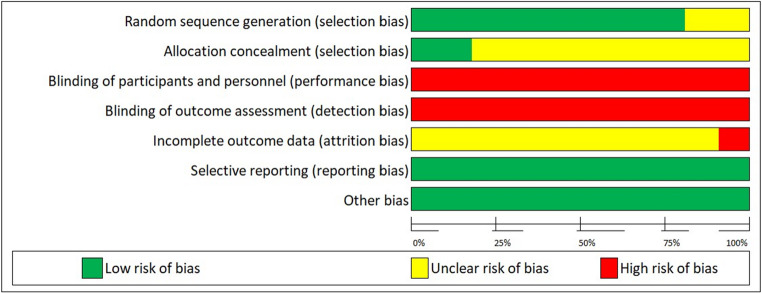
Risk of bias summary

 The risks of bias across all included studies are shown in Figure 3. Nine out of eleven RCTs adopted appropriate methods of randomisation. Allocation concealment was clearly described in nearly 50% of trials. However, none of the RCTs blinded participants, personnel, and outcome assessment. Most trials failed to explain the attrition rate or losses to follow. None of the RCTs has selective reporting or other biases.

###  Assessment of Transitivity and Consistency

 Participant characteristics, including mean age, BMI, and diabetes duration, were generally similar across studies, although some variability was observed (BMI<30 to >35 kg/m²; diabetes duration 1.5–10.6 years). All studies compared surgical procedures with medical or lifestyle management, which provided a well-connected treatment network. Results of global and local consistency assessments are presented in the Supplementary File 1: Assessment of consistency (global and local). No major inconsistency was detected across the network.

##  Primary Outcomes

###  Remission of T2DM

 For studies across mid-term follow-up durations, BPD (OR: -5.14 [95% CI -7.33 to -2.96]), LAGB (OR: -3.03 [95% CI -4.1 to -1.96]), RYGB (OR: -3.67 [95% CI -4.85 to-2.5]), SG (OR: -3.22 [95% CI -4.64 to -1.81]) were all significantly effective in comparison with MT at achieving the remission of T2DM (Table 2).

**Table 2:** League table of Mid-term efficacy of bariatric surgery types for T2DM: remission of T2DM and HbA1c

MT	SG	RYGB	LAGB	BPD
MT	-3.22 (-4.64, -1.81)	-3.67 (-4.85, -2.50)	-3.03 (-4.10, -1.96)	-5.15 (-7.33, -2.96)
-1.73 (-2.38, -1.09)	SG	-0.45 (-1.31, 0.41)	0.19 (-1.17, 1.56)	-1.92 (-4.04, 0.19)
**-1.88 (-2.09, -1.68)**	-0.15 (-0.78, 0.48)	**RYGB**	**0.64 (-0.44**,** 1.73)**	**1.47 (-3.41**,** 0.46)**
**-1.24 (-1.45, -1.04)**	0.49 (-0.16, 1.14)	**0.64 (0.44**,**0.84)**	**LAGB**	**2.11 (-4.29**,** 0.07)**
**-31.75 (-37.73, -25.77)**	**-30.02 (-36.03**,** -24.00)**	**-29.87 (-35.85**,** -23.88)**	**-30.51 (-36.49**,** -24.52)**	**BPD**

Results for Diabetes Remission are shown in the upper-right half of the matrix (e.g., odds ratios), and results for HbA1c are shown in the lower-left half (e.g., mean differences). Text in bold indicates statistical significance

 For studies with long-term follow-up periods, BPD (OR: -3.54 [95 CI -5.14 to -1.94]), RYGB (OR:-2.52 [95% CI -3.63 to -1.4]), SG (OR: -2.16 [95% CI -3.45 to -0.87]) were significantly superior to MT at achieving the remission of T2DM, except for LAGB (OR: -1.09 [95% CI -3.24 to 1.06]) (Supplementary File 1: Effect sizes - Figure 1) . 

 Based on the SUCRA values, BPD would appear to be the best surgery type for remission of T2DM. The SUCRA value for BPD was 1.00. Following this, RYGB (0.62) was second best, SG (0.55) was third best and LAGB (0.30) was fourth in rank (Supplementary File 1: SUCRA values - Table 1) .

###  HbA1c 

 In mid-term follow-up durations, BPD (MD: -31.75 [95% CI -37.73 to -25.77]), LAGB (MD: -1.24 [95% CI-1.45 to -1.04]), RYGB (MD: -1.88 [95% CI -2.09 to -1.68]), SG (MD:-1.73 [95% CI -2.38 to -1.09]) had a statistically significant effect in lowering HbA1c levels (Table 2).

 Likewise, for studies with long-term follow-up durations, BPD (MD: -1.89 [95% CI -3 to -0.77]), LAGB (MD: -1.06 [95% CI -1.75 to -0.38]), RYGB (MD: -1.79 [95% CI-2.49 to -1.09]), SG (MD: -1.87 [95% CI -3.03 to -0.71]) were significantly superior to MT in lowering HbA1c levels (Supplementary File 1: Effect sizes - Figure 2).

 BPD was observed to be the best surgical intervention for HbA1c with a SUCRA value of 1.00. After that, RYGB (0.67), SG (0.56), and LAGB (0.27) were ranked second, third, and fourth, respectively (Supplementary File 1: SUCRA values - Table 2) .

###  Secondary Outcomes

####  BMI

 For studies across mid-term follow-up durations in terms of BMI, LAGB (MD: -2.6, [95% CI -4.84 to-0.36]) and RYGB (MD: -5.88, [95% CI -7.49 to -4.28]) were highly effective in comparison with MT in lowering BMI levels.

 For studies across long-term follow-up durations, BPD (MD: -8.58, [95% CI -11.64 to -5.52]) and RYGB (MD: -5.05, [95% CI -5.84 to -4.24]) were significantly superior to MT in lowering BMI levels, except for LAGB (MD: -1.79, [95% CI-3.63 to 0.04]) (Supplementary File 1: Effect sizes - Figure 3) .

####  TG and TC

 In lowering TG across mid-term follow-up durations, only RYGB (MD: -62.61, [95%, CI -117.33 to-7.9]) is effective in comparison with MT. As compared with MT across long-term follow-up, differences in TG levels from baseline were as follows: BPD (MD: -34.12 [95% CI -52.79 to -15.49]), RYGB (MD:-18.3 [95% CI -32.01 to -4.59]), SG (MD: -49.47 [95% CI -76.01 to-22.93]). Therefore, BPD, RYGB, and SG reduced TG to a significantly greater extent than MT (Supplementary File 1: Effect sizes - Figure 4) . 

 In terms of TC, there were no statistical differences among BPD, RYGB, and LAGB across mid-term follow-up durations. In long-term follow-up durations, BPD (MD: -60.1 [95% CI -87.09 to -33.12]) and LAGB (MD: 8.44 [95% CI 3.92 to 12.97]) are significantly more effective than MT in lowering TC levels (Supplementary File 1: Effect sizes - Figure 5) . 

####  LDL

 For the LDL outcome, mid-term data were available only for LAGB and RYGB. Neither treatment showed a statistically significant difference compared to medical treatment (LAGB: MD 9.42 [95% CI -8.24 to 27.08]; RYGB: MD 2.45 [95% CI -10.76 to 15.66]).

 In the long-term follow-up, among all surgical interventions, only BPD demonstrated a statistically significant reduction in LDL levels compared to medical treatment (MD: -45.64 [95% CI -68.65 to -22.63]). In contrast, SG was associated with a statistically significant increase in LDL levels relative to medical treatment (MD: 16.49 [95% CI 0.63 to 32.35]) (Supplementary File 1: Effect sizes - Figure 6) .

####  SBP

 For the SBP outcome, mid-term follow-up data were available for LAGB, RYGB, and SG. However, none of these interventions demonstrated a statistically significant effect on SBP compared to medical treatment.

 Similarly, in the long-term follow-up (where BPD was also included), no surgical intervention resulted in a statistically significant reduction in SBP relative to medical treatment. Although some reductions were observed over the long term, these changes did not reach statistical significance (Supplementary File 1: Effect sizes - Figure 7).

####  DBP

 In mid-term follow-up, surgical procedures including LAGB, RYGB, and SG did not result in a statistically significant reduction in DBP compared to medical treatment.

In contrast, long-term data showed that RYGB significantly reduced DBP compared to medical treatment (MD: -2.55 [95% CI -4.78 to -0.32]). Conversely, LAGB was associated with a significant increase in DBP in the long term (MD: 5.55 [95% CI 1.57 to 9.53]). Although the overall trend across surgical interventions favoured DBP reduction, the remaining procedures did not demonstrate statistically significant effects when compared to medical treatment (Supplementary file 1: Effect sizes - Figure 8).

####  Adverse Events 

 Eight of the included studies reported adverse events in their publications. Definitions of adverse events varied across studies. Although no intraoperative deaths were reported, one patient developed a superficial wound infection at the access port site two weeks after placement, which resolved with intravenous antibiotics [26]. Another patient experienced eating difficulties and persistent regurgitation despite having no saline in the band and no evidence of obstruction on contrast study [26]. Mingrone et al. reported that one patient in the medical group died from a myocardial infarction during the 5-year follow-up period. Reported metabolic complications included iron-deficiency anaemia (n= 33, RYGB; n=2 BPD) [8, 23], vitamin B12 deficiency (n = 1, RYGB) [21], elevated parathyroid hormone levels (n = 1, RYGB) [21], hypoalbuminemia (n = 2) [23], and intravenous treatment for dehydration (n = 1) [24]. Specifically, hypoglycaemic episodes were reported in 32 of 50 patients in the RYGB group, 40 of 49 patients in the SG group, and 39 of 43 patients in the medical treatment group [8]. Gastrointestinal complications reported across the included studies included bowel obstruction (n = 1, RYGB; n = 1, SG; n = 1, MT) [24], ulcer formation (n = 4, RYGB) [24], dumping syndrome (n = 4, RYGB; n = 1, SG) [24], and acute pancreatitis (n = 6, RYGB) [21]. Microvascular and macrovascular complications included nephropathy (n= 7, RYGB; n = 5, SG; n = 4, MT) [24], retinopathy (n = 1, RYGB; n = 2, SG) [24], and stroke (n = 1, SG) [24]. Reported surgical complications included incisional hernia (n = 1, BPD) and intestinal occlusion (n = 1, RYGB) [23], as well as bariatric reoperations (n = 1, RYGB; n = 1, LAGB) [11].

##  Discussion

 In recent years, managing and preventing T2DM—the coexistence of obesity and type 2 diabetes—has emerged as a major global priority for healthcare providers [29, 30]. Although long-term weight loss can significantly help prevent and manage T2DM, achieving it remains difficult for many patients [29, 31]. In 2016, a joint statement from international diabetes organizations recognized bariatric surgery as a treatment option within diabetes management guidelines [32]. To date, this study is the first network meta-analysis of high-quality RCTs comparing the mid- and long-term outcomes of bariatric procedures versus MT on remission of T2DM, HbA1c levels, along with cardiometabolic outcomes (e.g., SBP, DBP, TC). In data from 740 people with T2DM, as compared with MT, all bariatric procedures (BPD, LAGB, RYGB, SG) resulted in significantly higher rates of T2DM remission at mid- and long-term follow-up durations and significantly higher rates of achieving lower HbA1c levels.

 Our results suggest that BPD appears to be the most effective bariatric surgery for achieving mid- and long-term T2DM remissions and HbA1c levels; however, empirical evidence remains limited. BPD was considered an advancement over RYGB due to its use of the distal 250 cm of the small intestine and a larger gastric pouch, often described as an ’eyeball-shaped’ stomach. However, the traditional BPD is primarily a malabsorptive procedure that have seen limited use over the past decade due to its technical complexity and high complication rates [33, 34]. Our data were supported by Ding et al. 2020 [15] and Harris et al. 2019 [35], in which the authors identified a better improvement in T2DM remission in the BPD group than in the RYGB group. Clinical evidence supports our findings that BPD is the most effective procedure for achieving HbA1c and T2DM remission. As a result, it should be primarily considered for people with T2DM and comorbid obesity, and managed with long-term follow-up in specialized medical centres [32]. However, we need more future RCTs focused on BPD to be conclusive as there was only one study in our network-analysis.

 Interestingly, we observed a lower rate of T2DM remission followed for 5 years or more compared to those followed between 2 and 5 years. This suggests a potential risk of diabetes returning in the long term after bariatric surgery. Indeed, recent research emphasizes that diabetes remission is both important and attainable, especially in its early stages. These findings demonstrate that bariatric surgery outperforms medical and lifestyle treatments, with remission rates of 51% at 1 year and 18% at 7 years in the surgical group, compared to just 0.5% at 1 year and 6% at 7 years in those receiving medical or lifestyle interventions [36]. Other studies have also noted a decline in diabetes remission rates over time following bariatric surgery, which may be linked to weight regain, the resolution of negative calorie balance, and gradual deterioration of β-cell function [36]. However, further long-term follow-up research is necessary to validate our findings. Even when relapse happens, people with T2DM who underwent bariatric surgery maintain better glycaemic control and require fewer medications [37, 38]. Additionally, other studies have shown that even short-term remission can provide benefits, with the risk of microvascular complications decreasing by an estimated 19% for each year of remission achieved [39]. These results suggest that bariatric surgery can be appropriately chosen regardless of a patient’s initial age and BMI not significantly associated with T2DM remission [16], though further research is needed to determine the most suitable type of bariatric surgery. 

 Our results suggest that BPD is the most effective surgery for achieving all secondary outcomes (BMI, TC, TG, LDL, and DBP, except SBP); however, there is a paucity of evidence supporting this claim. Lipid (TC, TG, LDL) and blood pressure (SBP, DBP) profiles were substantially improved after the BPD procedure, including reduced BMI, decreased TC, TG, LDL and DBP. These findings have been confirmed by further studies [40, 41]. In addition, RYGB leads to a significant improvement in BMI and TG levels, and provides some of the most significant long-term cardiovascular benefits, particularly for people with pre-existing risk factors [42]. However, there were no statistically significant differences in LAGB on lipid and blood pressure profiles. This confirms the results of previous network meta-analysis reporting a greater efficacy of RYGB than LAGB for BMI, with no differences in HbA1c levels and diabetes remission [43].

 Some limitations of the current network meta-analysis need to be acknowledged and taken into account when interpreting the findings. First, there is a limited number of RCTs with follow-up periods longer than two years. Specifically, only a few RCTs on BPD have been reported in the literature. Consequently, available data on diabetes remission and HbA1c, lipid, and blood pressure profiles are limited. The secondary limitation relates to the heterogeneity among studies, as they differ in their definitions of diabetes remission, types of bariatric surgery performed, geographic regions, follow-up durations, and the duration of diabetes in participants. Nevertheless, a random-effects model was applied when appropriate to provide the most cautious estimates. An additional limitation is that several studies involve small sample sizes, indicating that the combined results should be validated by further research. Lastly, differences in follow-up durations could have influenced the outcome measures. To address this, we performed subgroup analyses on studies with follow-up periods exceeding two years and five years, which yielded results consistent with the overall analysis. 

##  Conclusion

 In summary, moderate mid- and long-term effects of T2DM remission and HbA1c levels were observed after all surgical procedures. However, BPD appears to be the most effective surgery for achieving long-term diabetes remission, HbA1c levels, and lipid and blood pressure profiles; however, empirical data on this area is limited. RYGB is the most favourable option to manage HbA1c levels and BMI. The effects of other surgical types on BMI, lipid, and blood pressure are inconclusive and require further research. 

## Supplementary Information

Below is the link to the electronic supplementary material.

Supplementary Material 1 (DOCX 995 KB) 

## Data Availability

Data is provided within the manuscript or supplementary information files.

## Abbreviations

T2DM: Type 2 Diabetes Mellitus
HbA1c: Haemoglobin A1c
BMI: Body Mass Index
TG: Triglyceride
TC: Total Cholesterol
LDL: Low Density Lipoprotein
SBP: Systolic Blood Pressure
DBP: Diastolic Blood Pressure
