# Supplementary material for: Comparative Mid- to Long-Term Effects of Bariatric Surgery Versus Medical/Lifestyle Management in Type 2 Diabetes Mellitus: A Network Meta-Analysis of Randomized Controlled Trials
Source: Obes Surg. 2026 Jan 23;36(3):1304–14. doi: 10.1007/s11695-025-08451-x (PMC13038774; doi:10.1007/s11695-025-08451-x)
Supplement: Supplementary file 1 — Supplementary Material 1 (DOCX 995 KB) [file 11695_2025_8451_MOESM1_ESM.docx]

**Supplementary File 1**

**Search strategy**

Ovid MEDLINE(R) ALL <1946 to June 01, 2024>

1 exp Diabetes Mellitus, Type 2/

2 (non insulin$ depend$ or noninsulin$ depend$ or non-insulin$ depend$).tw,ot.

3 (NIDD or T2D or T2DM).tw,ot.

4 ((late or adult$ or mature$ or slow or stable$) adj3 onset).mp. and diabet$.tw,ot.

5 1 or 2 or 3 or 4

6 exp Bariatric Surgery/

7 (bariatric surger* or gastric surger* or metabolic surger* or bariatric surgical procedur*).tw,ot.

8 6 or 7

9 5 and 8

10 limit 9 to randomised controlled trial

**Assessment of consistency (global and local)**

**Remission**


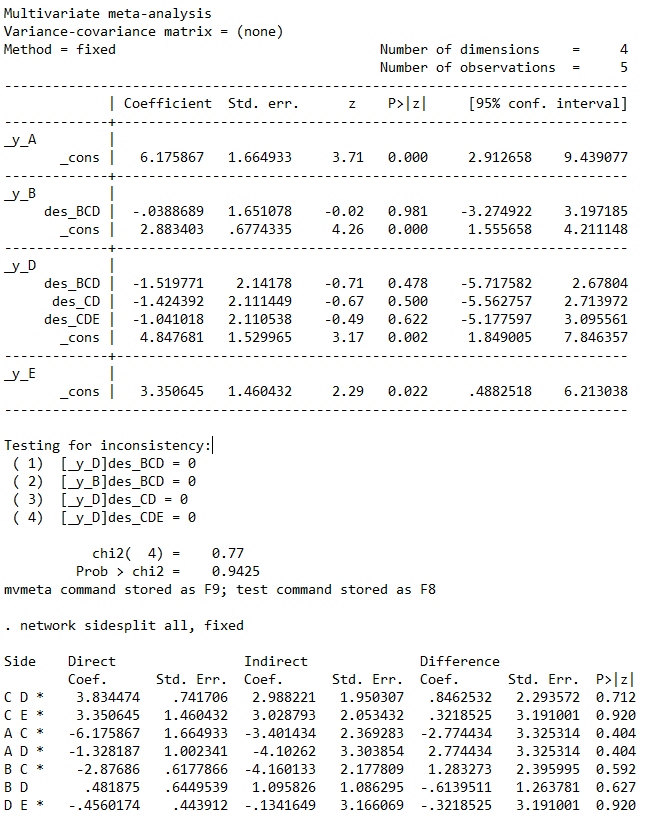


**HbA1c**


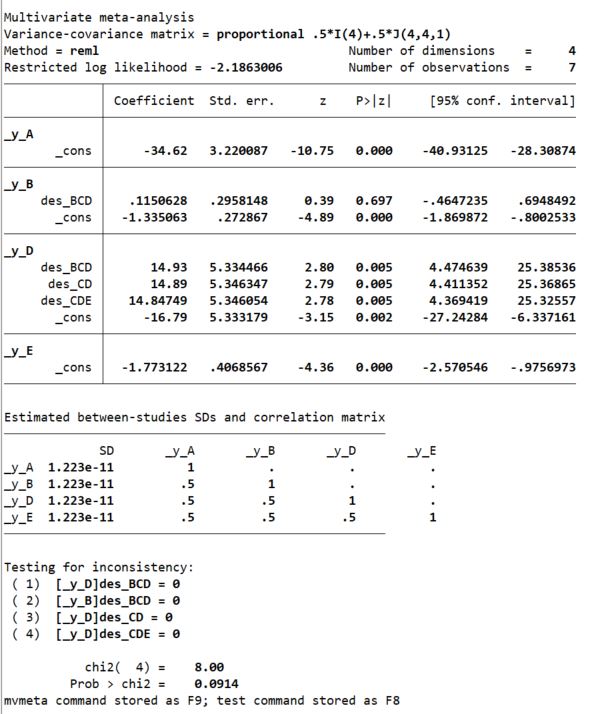


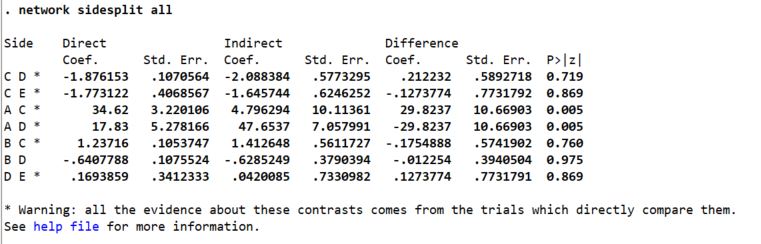


**BMI**


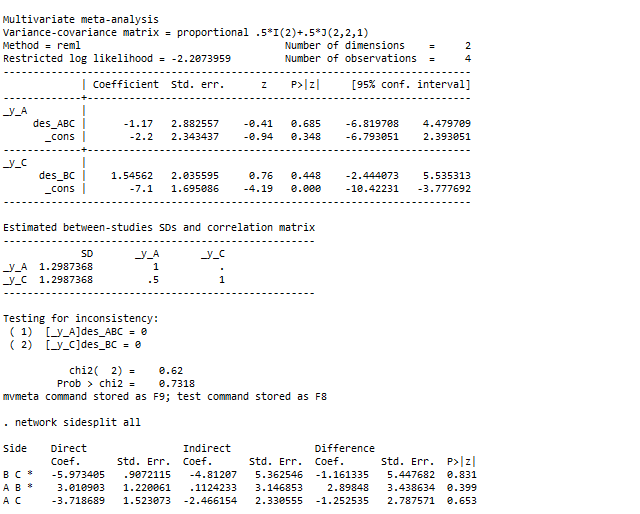


**TG**


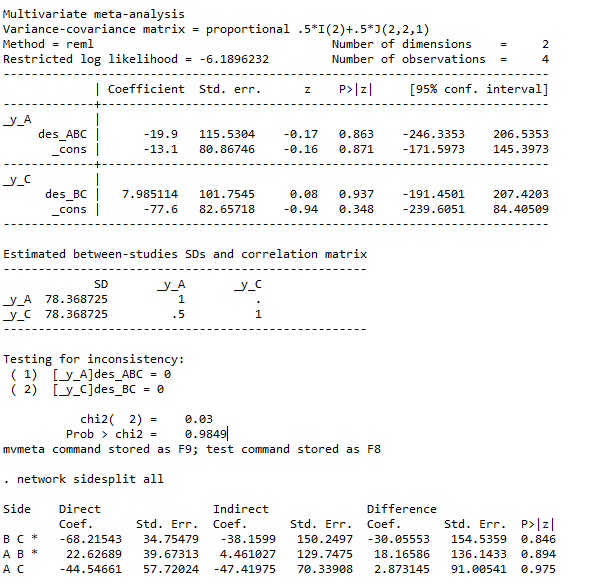


**TC**


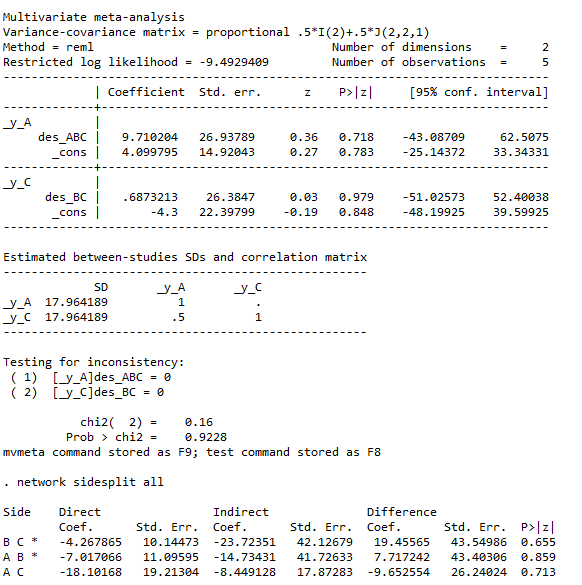


**LDL**


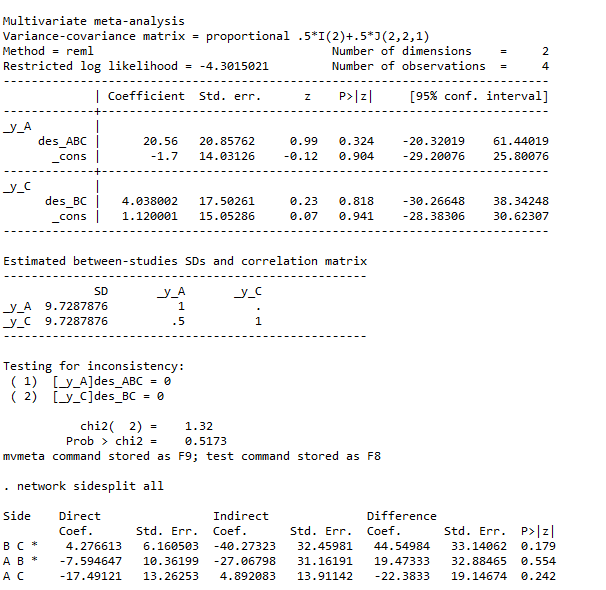


**SBP**


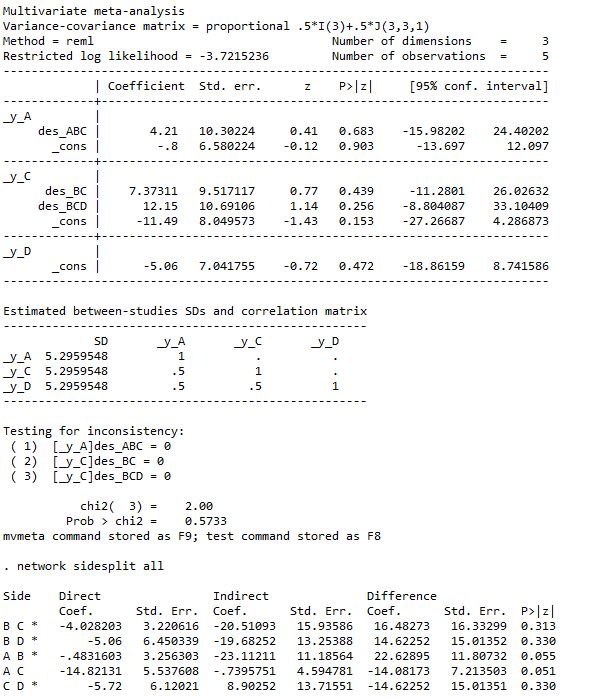


**DBP**


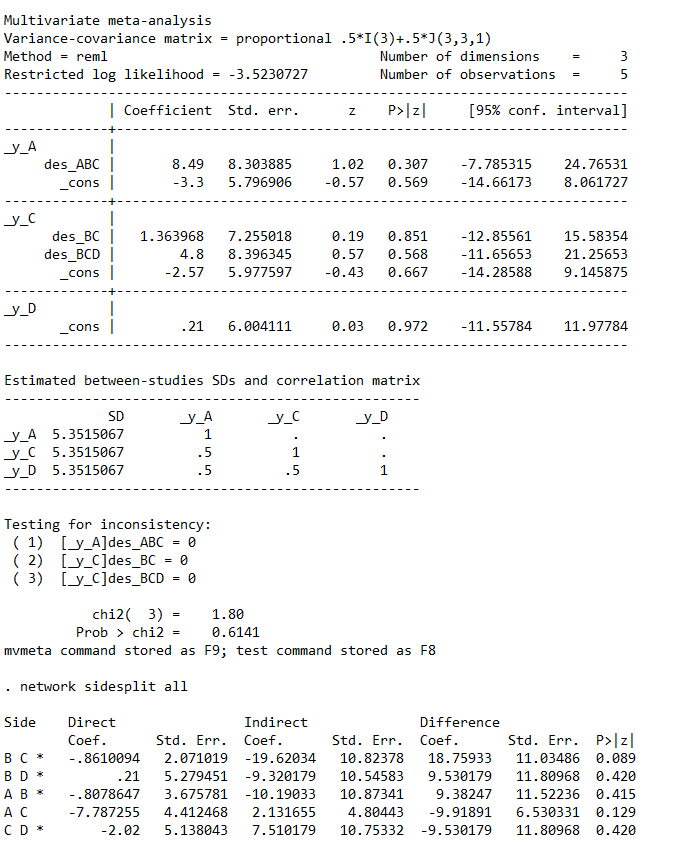


**Effect sizes**

**Figure 1. Remission of T2DM**


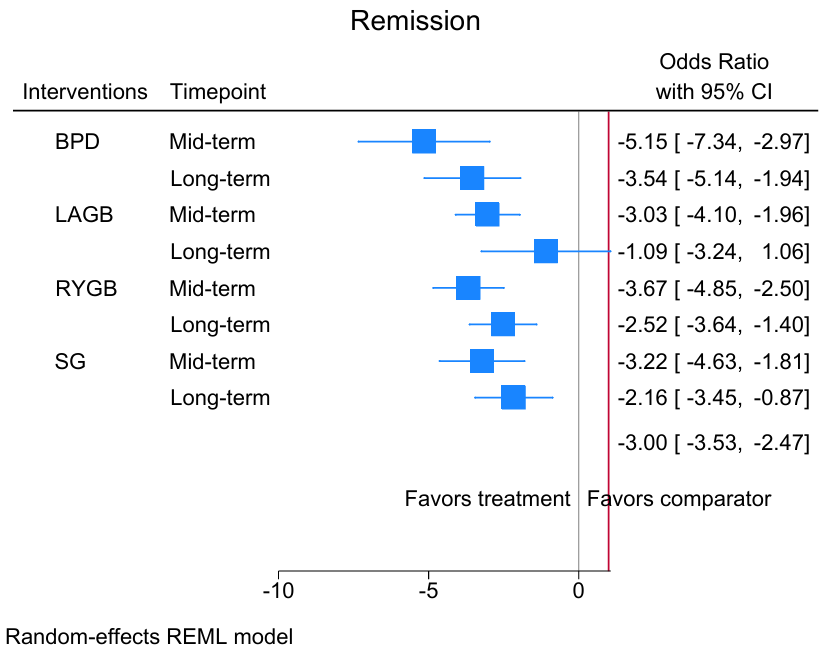


**Figure 2. Forest plot of HbA1C levels**


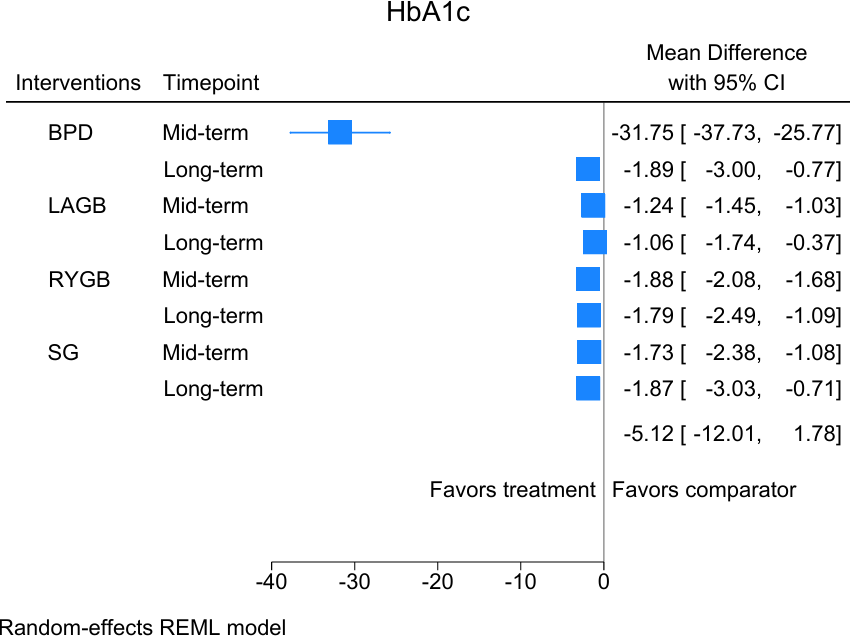


**Figure 3. Forest plot of BMI**


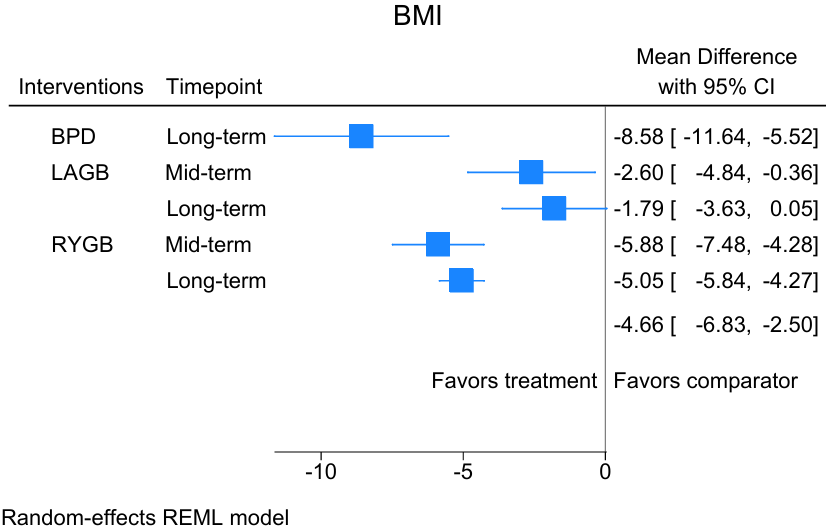


**Figure 4. Forest plot of triglyceride**


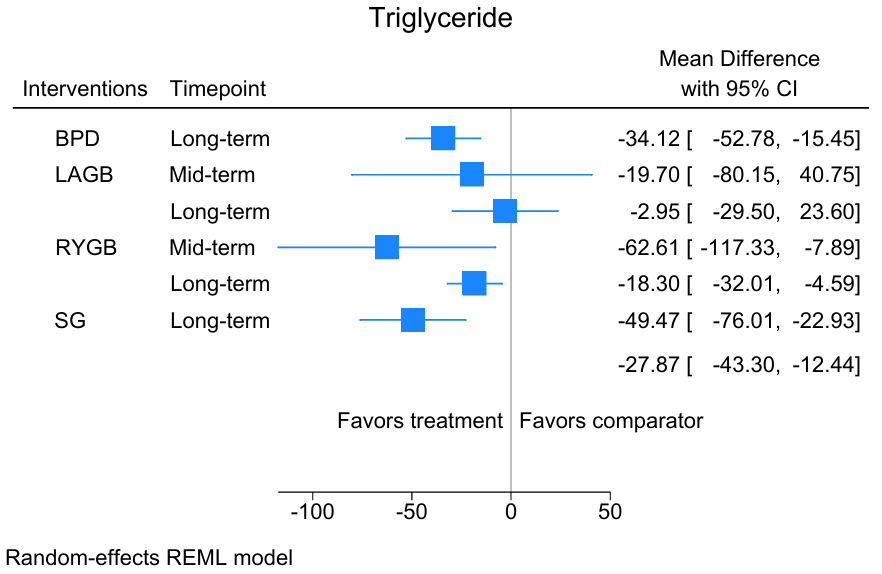


**Figure 5. Forest plot of total cholesterol**

**
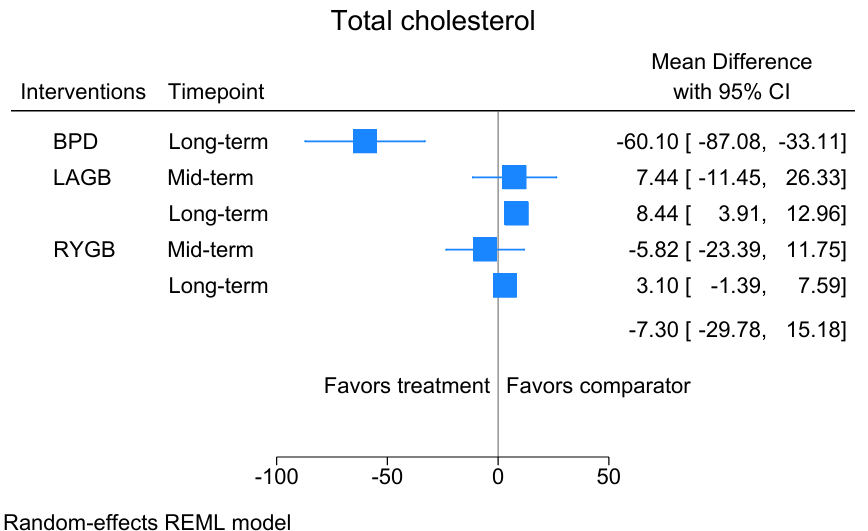
**

**Figure 6. Forest plot of LDL**


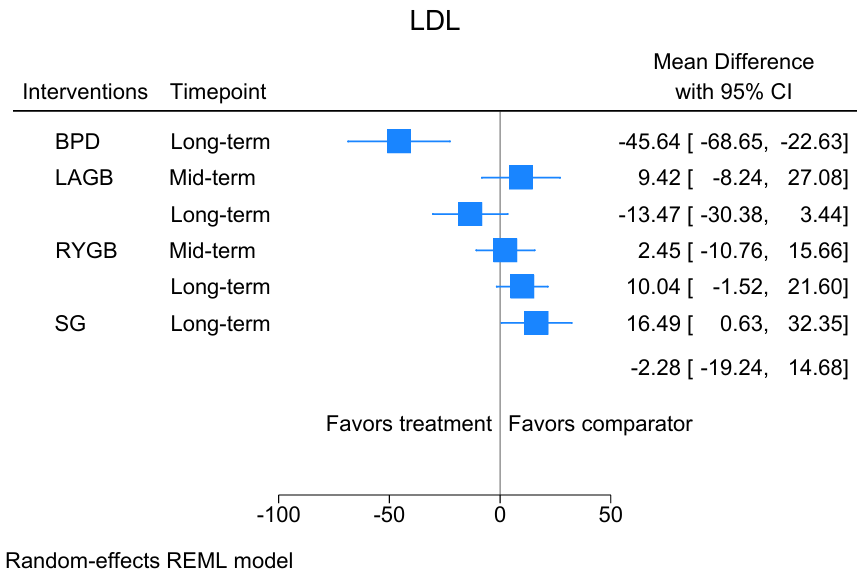


**Figure 7. Forest plot of SBP**


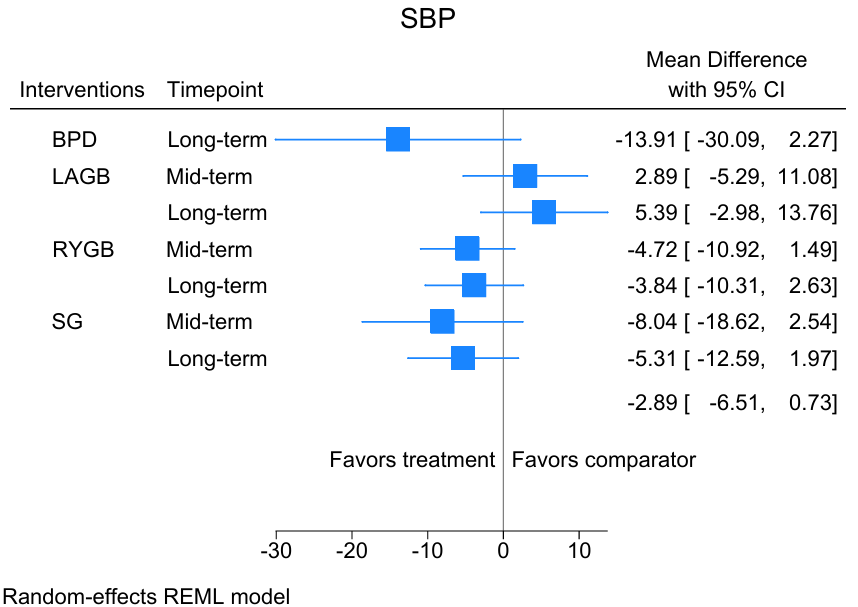


**Figure 8. Forest plot of DBP**


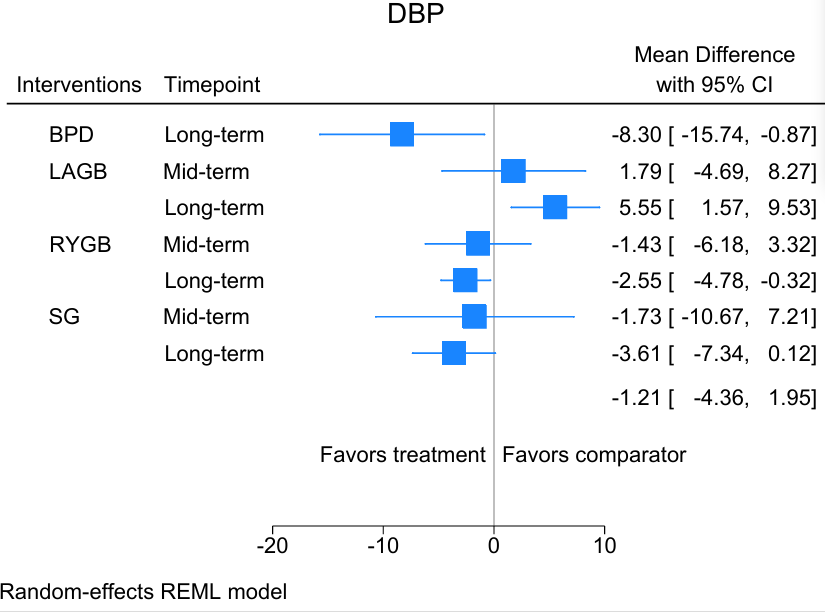


**SUCRA values**

**Table 1. Remission**

| **Intervention** | **SUCRA** |
| --- | --- |
| BPD | 1.00 |
| RYGB | 0.62 |
| SG | 0.55 |
| LAGB | 0.30 |
| MT | 0.03 |

**Table 2. HbA1c**

| **Intervention** | **SUCRA** |
| --- | --- |
| BPD | 1.00 |
| RYGB | 0.67 |
| SG | 0.56 |
| LAGB | 0.27 |
| MT | 0.00 |

**Table 3. BMI**

| **Intervention** | **SUCRA** |
| --- | --- |
| RYGB | 0.99 |
| LAGB | 0.50 |
| MT | 0.00 |

**Table 4. TG**

| **Intervention** | **SUCRA** |
| --- | --- |
| RYGB | 0.93 |
| LAGB | 0.44 |
| MT | 0.13 |

**Table 5. TC**

| **Intervention** | **SUCRA** |
| --- | --- |
| LAGB | 0.80 |
| MT | 0.51 |
| BPD | 0.19 |

**Table 6. LDL**

| **Intervention** | **SUCRA** |
| --- | --- |
| LAGB | 0.75 |
| RYGB | 0.57 |
| MT | 0.18 |

**Table 7. SBP**

| **Intervention** | **SUCRA** |
| --- | --- |
| SG | 0.88 |
| RYGB | 0.70 |
| MT | 0.30 |
| LAGB | 0.12 |

**Table 8. DBP**

| **Intervention** | **SUCRA** |
| --- | --- |
| RYGB | 0.67 |
| SG | 0.65 |
| MT | 0.45 |
| LAGB | 0.24 |
